# 592. Burn-specific Skills Fair Improves Nursing Staff Confidence

**DOI:** 10.1093/jbcr/irag033.386

**Published:** 2026-04-06

**Authors:** John Gualano

**Affiliations:** Loyola University Medical Center, Aurora, IL

## Abstract

**Introduction:**

Our burn unit was faced with the unique challenge of adhering to burn-specific domain requirements and critical care/clinical skills that are expected of a specialty ICU. Lacking was specialty ICU-focused delivery of hands-on education, domain organization, and staff assessment of perceived needs. In an attempt to equally distribute and incorporate American Burn Association (ABA) domains into clinical skill education while maintaining confidence amongst a growing cohort of new-grad/new-hires, a regularly spaced skills fair was established. The fair was led by experienced peers with multi-disciplinary involvement and reference, ultimately aimed at improving nursing confidence and knowledge of tested domains.

**Methods:**

Burn unit-based council (UBC) met monthly to establish quarterly skill fair dates, determine cluster of stations utilizing a frequency tracker, establish validators, develop competency sign-offs, and assess data. Validators utilized, collaborated with, and referenced an applicable unit resource when creating their station content: Surgeons, Nurse Practitioners, Pharmacist, Dietitian, Respiratory Therapy, Physical/Occupational Therapy, Social Work, Advanced Burn Life Support manual, policies, or protocols.

Principles of active recall and spaced repetition were roughly followed with a goal of re-visiting a competency before its year-mark, utilizing a heat map/frequency tracker of progressing color schemes to identify when a skill was overdue or, conversely, highlight when a skill had consecutive fair validations to allow for easy-to-follow visualization.

A subjective Likert Scale (1-5) was implemented over two quarters to gauge staff confidence amongst greatly varied experience levels.

A knowledge-based questionnaire was added for each domain testing un-biased recall.

The post-skill assessment and survey results allowed for customizable station content and frequency based on unit need. This also allowed staff to request preferred content delivery, strategies for engagement, specific skills, and multi-disc collaboration.

**Results:**

Subjectively, staff demonstrated an increased mean scoring for confidence across all domains, with respective decreases in standard deviation, demonstrating a greater consistency. Objectively, staff demonstrated an increased knowledge-based questionnaire mean from pre- to post-testing across all domains.

**Conclusions:**

Standardization of material, spaced repetition, and implementation of pre- and post-testing has improved staff knowledge and confidence, and has streamlined our ongoing competency challenges.

**Applicability of Research to Practice:**

Identifying the unique training needs of specialized critical care nurses prompted a method for repeated delivery, assessment of competency, and experience considerations. Implementation of similar pre- and post-assessments for subjective and objective testing can aid specialty ICU training and guide validation methods.

**Funding for the study:**

N/A.

